# Impact of pharmacist-led microbiology result follow-up post-discharge for patients undergoing inpatient infectious diseases consultation

**DOI:** 10.1017/ash.2024.348

**Published:** 2024-08-07

**Authors:** Amy L. Van Abel, Abinash Virk, Kristin Cole, Trudi Lane, Douglas Osmon, Margaret Pertzborn, Diana Schreier, Hilary Teaford, Courtney Willis, Anna Woods, Christina G. Rivera

**Affiliations:** 1 Department of Pharmacy, Mayo Clinic, Rochester, MN, USA; 2 Division of Public Health, Infectious Diseases, and Occupational Medicine, Mayo Clinic, Rochester, MN, USA; 3 Department of Pharmacy, Mayo Clinic Health System—Northwest Wisconsin Region, Eau Claire, WI, USA; 4 Department of Pharmacy, Mayo Clinic, Jacksonville, FL, USA

## Abstract

**Objective:**

The primary objective was to determine the rate of clinical actions taken post-discharge on updated microbiology results by an ID pharmacist-led team. Secondary objectives were to describe the microbiology results requiring intervention, characterize interventions by type and severity, and determine time from result to clinical review.

**Design:**

Retrospective cohort study.

**Setting:**

Four hospitals within Mayo Clinic, including two large academic centers and two Mayo Clinic Health System sites.

**Participants:**

Adult patients at four sites within Mayo Clinic from 1/1/2019 to 2/28/2023. Eligible patients had a hospitalization with an ID consult and an updated microbiology result reported after discharge.

**Intervention:**

Pharmacists reviewed a report of selected patients with microbiology tests that resulted post-discharge within the last 24–96 hours. Interventions were recorded electronically in real-time by the pharmacist. Of those patient encounters with an intervention, a sample of 200 patient encounters was randomly selected for detailed chart abstraction.

**Results:**

A total of 6,792 encounters with at least one microbiology result reviewed post-discharge were identified. Of these encounters, 1977 (29%) had at least one resulting intervention. Median time from test update to clinical review was 27.2 hours (IQR 21.6–69.6). The highest severity ratings, in which failure to intervene may have resulted in patient harm, were assigned to the intervention in 28% of cases.

**Conclusions:**

For patients seen by an inpatient ID consult service, a post-hospital discharge microbiology result review process performed by ID-trained pharmacists effectively addressed abnormal results during the transition of care. Similar processes may be considered at other institutions.

## Introduction

Transitions of care (TOC) have emerged as an important vulnerability point in the healthcare system where medical errors and clinical deterioration can occur.^1^ A common gap in TOC from an acute hospitalization is follow up on pending inpatient testing. It has been estimated that as many as 41% of patients may have pending test results at the time of hospital discharge, and up to 9.4% of these may be clinically actionable.^2^ Pending microbiology testing can be prone to this scenario, as certain microbiology results can take days to weeks to finalize. These late microbiology results may include serologies and cultures such as anaerobic, mycobacterial, or fungal. One study found about half of patients discharged from the emergency department on antibiotics needed therapy modification after culture and susceptibilities results returned.^3^

Post-discharge culture and susceptibility review has been successfully utilized in emergency departments (ED) for over a decade. Dedicated time to perform culture review by physicians may be limited, thus clinical pharmacists have also been leveraged for this task. Antimicrobial regimen modification rate and time to review were similar between pharmacists and physicians for ED post-discharge cultures, while readmission rates were lower with pharmacist ED culture review.^2,3^ Literature also supports a similar role for pharmacist culture review in the urgent care (UC) setting.^4^ In 2017, Mayo Clinic Rochester instituted a post-discharge microbiology test monitoring program for patients with an inpatient Infectious Diseases (ID) consult during acute care hospitalization which involved result review by ID-trained ambulatory care pharmacists. The process was extended to three other regional sites several years later. Pharmacist(s) review a custom electronic medical record (EMR) report of inpatient ID consult patients with microbiology result(s) that resulted post-discharge as one task along with other antimicrobial stewardship or outpatient antimicrobial therapy duties. Microbiology results were triaged to an ID physician or advanced practice provider (APP) for further review per the pharmacist discretion and operational workflow at each practice site. Interventions were recorded in real-time using a report functionality by the pharmacist. The primary aim of this study was to determine the utility and clinical impact of post-discharge updated microbiology result review for patients receiving an inpatient ID consultation. Secondary aims were to describe the post-discharge updated microbiology results requiring intervention, characterize the interventions by type and severity, and to determine time from result to clinical review.

## Methods

### Settings and participants

This retrospective study included adult patients (>18 years of age) at four Mayo Clinic hospital sites from January 1st, 2019, through February 28th, 2023. Mayo Clinic Rochester conducted the ID-trained pharmacist-led post-discharge updated microbiology review process throughout the entirety of the study period. The process was initiated at the other sites at different time points after the start of the study period with Mayo Clinic—Florida beginning on May 1st, 2022, Mayo Clinic Health System-Mankato beginning on October 1st, 2022, and Mayo Clinic Health System—Eau Claire beginning on Dec 1st, 2022. Specialty training of participating pharmacists included completion of a post-graduate year 2 (PGY-2) ID pharmacy residency, board certification in ID pharmacy, and/or on the job training as ID clinical pharmacy specialists. Learners on rotation in the ID clinic also participated in culture review under the direction of ID-trained pharmacists.

Eligible patients had a hospitalization of inpatient or observation class from which they were discharged alive, had an inpatient ID consultation in the prior 90 days, and had a microbiology result obtained by any inpatient provider from the hospital encounter that updated after hospital discharge or within the 24 hours just prior to discharge. All microbiology results were included other than urine cultures. Patients without a Minnesota research authorization were excluded. This study was deemed exempt by Mayo Clinic Institutional Review Board (IRB #22-012757).

### Operational process

Throughout the study period, these updated microbiology results were identified using an operational report within Epic (Epic, Verona, WI) that was reviewed by an ambulatory ID pharmacist every weekday during hours of normal ID clinic operation. The report provided one patient record to review per qualifying patient hospital encounter where an inpatient ID consult occurred in the past 90 days and had an abnormal microbiology result that was updated in the lookback period specified by the specific report version. Only microbiology tests obtained within the prior 42 days were evaluated due to report performance considerations, though it was expected results would be finalized by this time. The report considered an update as any change to a reported microbiology result, including but not limited to serologic result, growth or identification of new organisms, changes to susceptibility results, or new comments. Urine cultures were excluded from the report due to historical experience of high frequency and low/no clinical relevance to the ID consult matter in the post-discharge time frame.

Versions of the report existed for each specific site, and were available with one-day, three-day, and seven-day lookbacks to ensure result updates occurring over a weekend or holiday would be evaluated. While inpatient ID team members leveraged the same report for review of microbiology results of hospitalized patients, the report used in this study was pre-filtered for pharmacists to only discharged patients requiring review.

The pharmacist would document within the patient’s medical record if the result updates required an intervention and/or input or action from other ID team members (ID consultant, fellow, or APP). Following the pharmacist’s independent review of a specific patient encounter, regardless of whether an additional intervention was required, the pharmacist would select a button at the top of the report to timestamp their review of the patient. Upon this selection, a visual indicator column would change to a “review complete” status and the patient would subsequently no longer appear on the pre-filtered view upon report rerun. If a subsequent result update occurred, the visual indicator would change back to a “needs review” status and the patient would be included in the next report run for additional review.

### Outcomes

The primary study outcome was the percentage of updated abnormal microbiology results requiring pharmacist intervention. Secondary outcomes included time from result update to pharmacist review, pharmacist intervention types, pharmacist intervention severity, microbiology result type, and type of organism identified.

### Data collection

The details of pharmacist-initiated interventions based on the updated microbiology result review and demographic information were identified by retrospective electronic medical record (EMR) based reports. A random sample cohort of 200 pharmacist interventions was used for data abstraction on most of the secondary outcomes as outlined in Table 1. Each region was represented in this random sample in proportion to its representation in the larger cohort. Pharmacist intervention severity ratings were determined by researchers at the time of data collection using a scale modified from the National Coordinating Council for Medication Error Reporting and Prevention (NCC MERP) Index for categorizing medication errors.^5^ Severity ratings were defined as follows: Category 1—failure to intervene may have resulted in significant patient harm including death or permanent damage (eg, daptomycin-nonsusceptibility of *Staphylococcus aureus* in a patient discharged on daptomycin for *S. aureus* bacteremia); Category 2—failure to intervene may have resulted in minor or temporary patient harm (eg, identification of additional organisms in a polymicrobial infection, not covered by the current treatment regimen); Category 3—intervention resulted in therapy optimization such as de-escalation or decreased costs. All category 1 severity ratings were validated by a second researcher.

**Table 1.** Outcome assessment

	Population assessed	
	Entire cohort (*n* = 6792)	Sample of RPh interventions (*n* = 200)
**Primary outcome**		
% of abnormal patient encounters requiring RPh intervention	x	
**Secondary outcomes**		
Time from result update to RPh review	x	
RPh intervention types		x
RPh intervention severity		x
Microbiology result type		x
Type of organism identified		x
**Patient characteristics**		
Age	x	
Gender	x	
Race	x	
Length of hospital stay	x	
Type of infection		x

Abbreviations: RPh-pharmacist.

### Statistics

Data was summarized using means and standard deviations (SD) or medians and interquartile ranges (IQR) for continuous data and counts and percentages for categorical data. A 95% binomial exact confidence interval (CI) was calculated for the intervention rate. Logistic regression was used to assess the association between patient/hospital characteristics and need for an intervention. Associations were summarized using odds ratios (OR) and 95% CIs. All analyses were performed using SAS version 9.4 software (SAS Institute, Inc.; Cary, NC).

## Results

A total of 6,792 patient encounters with at least 1 microbiology result reviewed post-discharge were identified. Median time from abnormal microbiology result update to pharmacist review was 27.1 hours (IQR 21.6–69.6). Patients were predominantly white, and most patient encounters were from the Rochester region as other sites were not involved during the entire study period. Additional patient demographics are displayed in Table 2. Skin and soft tissue infections (including surgical site infection) represented the most common infection type associated with late microbiology result. Over 75% of all new microbiology results represented growth and identification of a new organism on culture, predominantly Gram-positive aerobic organisms as well as anaerobic organisms. Further infection characteristics are displayed in Table 3.

**Table 2.** Patient demographics (total cohort)

	Total (N = 6792)
**Age**, Mean (SD)	59.7 (15.9)
**Sex**	
Female	2727 (40.2%)
Male	4064 (59.8%)
Nonbinary	1 (0.0%)
**Race/ethnicity**	
Asian	129 (1.9%)
Black or African American	269 (4.0%)
Hispanic or Latino	257 (3.8%)
White	5919 (87.1%)
Other	153 (2.3%)
Unknown	65 (1.0%)
**Region**	
Florida Region	444 (6.5%)
MCHS NW WI Region	39 (0.6%)
MCHS SW MN Region	77 (1.1%)
Rochester Region	6232 (91.8%)
**LOS (days)**, Median (IQR)	5 (3, 9)
**Discharge disposition**	
Acute Care Hospital	56 (0.8%)
Home or Self Care	4420 (65.1%)
Home-HealthCare Svc	1430 (21.1%)
Hospice	116 (1.7%)
Left Against Medical Advice/Discontinued Care	40 (0.6%)
Rehab Facility	77 (1.1%)
Skilled Nursing Facility	567 (8.3%)
Transitional Care Unit	37 (0.5%)
Other	49 (0.7%)

Abbreviations: IQR—interquartile range, LOS—length of stay, MCHS—Mayo Clinic Health System, SD—standard deviation.

**Table 3.** Infection characteristics (focused review subset)

	Total (N = 200)
**Infection type** *	
Skin and soft tissue infection	43 (21.5%)
Intra-abdominal infection	30 (15%)
Pneumonia	27 (12.5%)
Osteomyelitis	25 (12.5%)
Prosthetic joint infection	21 (10.5%)
Bacteremia	18 (9%)
Urinary tract infection	7 (3.5%)
CNS infection	6 (3%)
Diabetic foot infection	4 (2%)
Other infection type	22 (11%)
**ID consult service specialty**	
General ID	87 (43.5%)
Orthopedic ID	57 (28.5%)
Transplant ID	14 (7%)
Hematology/oncology ID	25 (12.5%)
Intensive Care Unit ID	14 (7%)
**Microbiology result type** *	
Organism identification	151 (75.5%)
Susceptibility result—agar dilution	28 (14%)
Susceptibility result—automated	12 (6%)
Polymerase Chain Reaction result	11 (5.5%)
Gram stain	10 (5%)
Antibody testing	1 (0.5%)
Other	2 (1%)
**Type of organism associated with abnormal result** *	
Aerobic gram-positive bacteria	76 (38%)
Anaerobic bacteria	58 (29%)
Aerobic gram-negative bacteria Fungal—yeast Fungal—mold Mycobacteria Other	31 (15.5%) 25 (12.5%) 21 (10.5%) 11 (5.5%) 3 (1.5%)
**Specimen source** *	
Sputum Bone Tissue Aspirate Tissue swab	20 (10%) 9 (4.5%) 70 (35%) 1 (0.5%) 21 (10.5%)
Blood	19 (9.5%)
Body fluid	44 (22%)
Joint aspirate	3 (1.5%)
Bronchoalveolar lavage Drainage Prosthetic material Bronchial washing Other	5 (2.5%) 3 (1.5%) 7 (3.5%) 2 (1%) 5 (2.5%)

Abbreviations: ID—Infectious Diseases

* Sums >200 due to results in multiple categories per patient encounter

Of the 6,792 patient encounters, 1977 (29.1%) had at least one pharmacist intervention (95% CI, 28.0–30.2). A small subset of patient encounters had ≥2 interventions (*n* = 167; 8.5%). The remainder were independently reviewed by pharmacists and judged not to need further action.

**Figure 1.**
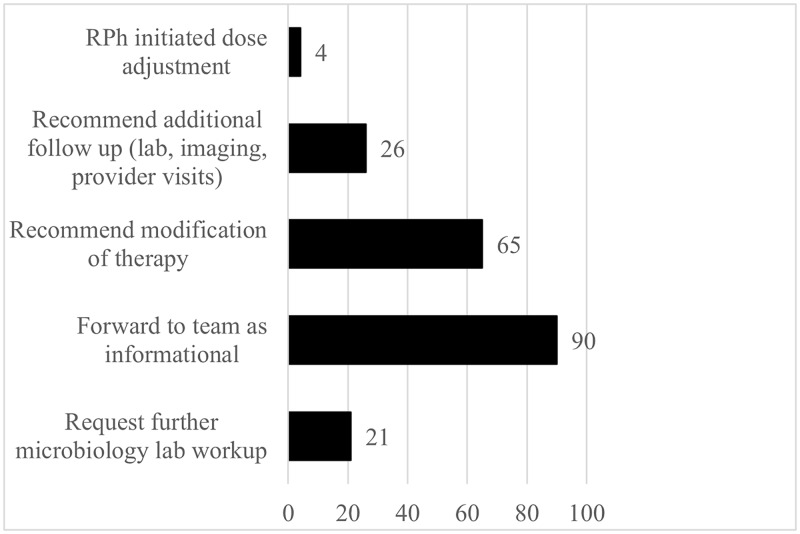
Pharmacist intervention types (*n* = 200).* *Sum >200 due to multiple intervention types per patient encounter.

**Figure 2.**
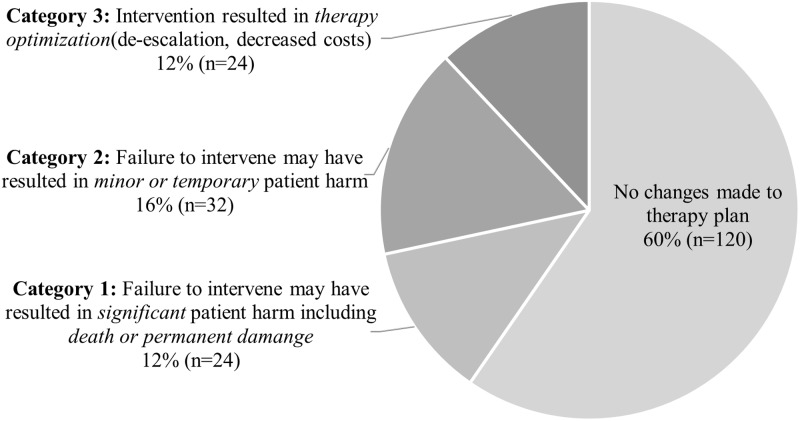
Intervention severity (*n* = 200).

A random sample cohort of 200 pharmacist interventions was further analyzed. The most common intervention was facilitation of result review by the ID team (ID consultant, fellow, or APP) as per ID sign off note, followed by recommendation of therapy modification. Intervention types and frequency are displayed in Figure 1. Modification of the therapy plan (medication, dose, labs, or other follow-up) occurred in 38% of cases in which there was an intervention. Certain pharmacist interventions were performed independently via collaborative practice agreement at applicable sites as described in Table 4. Suggestions for consideration of a change in the antimicrobial agent(s) resulted in therapy change in 53.6% of cases. Intervention severity ratings are depicted in Figure 2. In 12% of cases with pharmacist intervention, failure to intervene was deemed to have potentially resulted in significant patient harm, while in 16% of cases, it may have led to minor or temporary harm.

**Table 4.** Pharmacist scope of practice by CPA^1^ summary

ID pharmacist authority per CPA^1^	Common applications to microbiology result follow-up program
Evaluate the antimicrobial treatment regimen based on laboratory and microbiologic data	Independent pharmacist initial review of microbiology results Pharmacist review only of non-actionable results
Order and interpret medication labs and other tests related to the use of antimicrobials	Order/interpret antimicrobial susceptibility testing Bacterial Fungal Mycobacterial Order/interpret blood cultures Counsel patients on pertinent microbiology results as relevant
Assess and modify antimicrobial prescriptions	Adjust doses of antimicrobials (ie, increase daptomycin dose for SDD susceptibility result) Adjust duration of antimicrobial (ie, increase duration)
Issue new prescriptions and authorize refills	Discuss new or discontinuation of antimicrobial drug with ID physician or APP, then subsequently: Issue new antimicrobial prescription Discontinue antimicrobial prescription Counsel patients on the above antimicrobial changes
Order healthcare clinician consults after discussing with ID team member	Order and arrange for outpatient ID follow-up visits Physician APP Pharmacist
Document patient care and treatment decisions in the EHR	Document interventions in iVENT system Place EHR progress notes on: Interventions involving direct patient care Select highly clinically relevant microbiology results

Abbreviations: APP—Advanced Practice Provider; CPA—collaborative practice agreement; EHR—electronic health record; ID—Infectious Diseases; SDD—susceptible dose dependent.

1 Applicable at Minnesota and Wisconsin sites.

**Figure 3.**
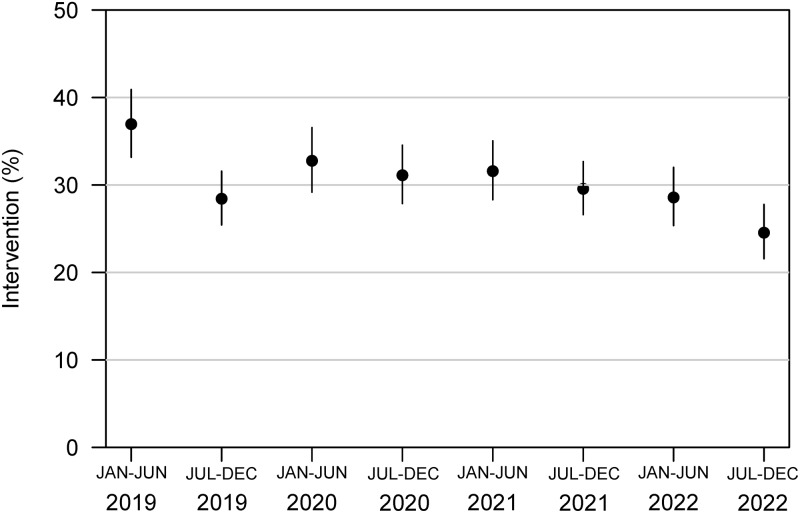
Intervention rates over time in Rochester.* *Per year, OR = 0.91, 95% CI, 0.86–0.95, *p* < 0.001.

Patient encounters with a longer inpatient length of stay were significantly less likely to require post-discharge pharmacist intervention (per day, OR = 0.97, 95% CI, 0.97–0.98, *p* < 0.001). Characteristics associated with higher rates of pharmacist intervention included male gender (OR = 1.16, 95% CI, 1.04–1.29, *p* = 0.007), admission at a large academic medical center site (OR = 2.22, 95% CI, 1.79–2.78, *p* < 0.001), and discharge to home rather than to another facility (OR = 1.49, 95% CI, 1.25–1.82, *p* < 0.001). Intervention rates at the largest tertiary medical center in Rochester decreased over time, as depicted in Figure 3 (per year, OR = 0.91, 95% CI, 0.86–0.95, *p* < 0.001).

## Discussion

While there is well established evidence for culture review results post-emergency department discharge, this is one of the very few studies that looks at post-hospital discharge review of pending microbiology tests.^1–3^ One small prospective study described the impact of post-discharge culture review by antimicrobial stewardship pharmacists for 38 patients. Five patients were prescribed a potentially inappropriate antimicrobial agent, and the pharmacist intervened in three of these cases (7.9% intervention rate). When compared to the pre-intervention period, inappropriate outpatient antimicrobial therapy was reduced by 39%.^6^ In our study, the pharmacist intervention rate was about three times as high which further supports the importance of a post-discharge microbiology follow-up and the effectiveness of ID-trained pharmacists having a role in the process. A reason for our observed higher intervention rate may be that pharmacists performed additional actions beyond just recommending an antimicrobial change, such as identifying additional microbiologic testing, antimicrobial dose adjustments, need for ID clinic follow-up visit, etc. The interventions in this study are in addition to existing safety features such as phone calls to providers for resulted deemed “critical,” and routine result routing to the ordering provider. The high intervention rate illustrates that review by ID-trained pharmacists may allow for better interpretation and patient care optimization as compared to review by non-ID-trained clinicians.

Our intervention rate results are also higher than those looking at finalized cultures post-ED discharge, with between 9% and 22% of culture results being clinically actionable.^1,4,6^ Our intervention rate of 29% was also higher than the 20% intervention rate by ID pharmacists documented in an exclusively ambulatory setting by Wattengel et al.^7^ This may be because hospitalized patients have a higher magnitude and more diverse microbiology tests including serial cultures obtained during their hospitalization as opposed to patients in an emergency department or ambulatory setting. However, patients with a longer length of hospital stay were less likely to require post-hospital discharge intervention, as likely most microbiology results finalized while the patient was admitted and there is a parallel process for microbiology result review by ID APPs for results that are updated between the time of ID sign off and hospital discharge. Our study also uniquely provides a severity rating to the interventions, illustrating this process results in patient harms avoided. Our efforts also may reduce readmission rates for antimicrobial therapy-related problems due to suboptimal or inappropriate therapy, similar to the findings by Randolph et al.^1^

Our study was quite large comparatively, given we were able to track interventions over 4 years at different sites in a health system. These sites included two large academic medical centers and two smaller health system hospitals, increasing generalizability. As the patients all had an ID consult, the types of cultures and microbiologic testing reviewed include a range of traditional microbiology (Gram stain and culture) to advanced testing such as broad range polymerase chain reaction with sequencing, demonstrating that microbiology follow-up programs have high yield for patients with all types of pending microbiology and infections. The general infection complexity in this study is higher than most or all other post-discharge culture monitoring programs described in the literature to date; however, ID-trained pharmacists were effective key facilitators of the process in collaboration with the multidisciplinary team. We were also able to utilize a pharmacist collaborative practice agreement at three sites to efficiently implement interventions. Advanced practice providers or inpatient based pharmacists may also be leveraged for culture review of ID consult patients.

As with any retrospective study, there is a potential for selection bias and dependence on accurate documentation. Our study was conducted within one health system, with a specific EMR, which may limit generalizability and applicability to those without the same EMR or resources to support reporting development. Additionally, the severity rating scale is inherently subjective. In an attempt to account for this, outcomes of Category 1 severity interventions required two reviewers to be in agreement. Furthermore, post-discharge microbiology result responses before implementing the Epic custom report was not investigated as a comparative group.

This study investigates the impact of post-hospital discharge microbiology result review by ID pharmacists, a less-explored area compared to post-ED discharge. Conducted over 4 years across multiple sites within a health system, the research involved pharmacists with ID training, leading to interventions in 29% of cases. Notably, more than 1 in 10 of patients faced potential significant harm, emphasizing the role of pharmacist-driven microbiology review in preventing harm and potential benefits on patient outcomes and readmission rates. Future studies should further investigate the impact of trained pharmacists or APPs on post-hospital microbiology results, focusing on clinical impact beyond intervention severity, including but not limited to repeat healthcare contact, adverse events, and physician time saved. For patients seen by an inpatient ID consult service, a post-hospital discharge microbiology result review process performed by ID pharmacists effectively addressed abnormal results during the transition of care. Similar processes may be considered at other institutions.
